# Understanding Diabetes Distress: A Study of Contributing Factors Among Adults With Type 2 Diabetes in Rural East Godavari, Andhra Pradesh

**DOI:** 10.7759/cureus.104183

**Published:** 2026-02-24

**Authors:** Ratna Jyothi Meka, Shanmuga Sundaram M, Geetha Rani K S, Vadivel Mani, Muninathan Natrajan

**Affiliations:** 1 Department of Physiology, Sapthagiri Institute of Medical Sciences and Research Centre, Bengaluru, IND; 2 Department of Anatomy, Farookh Academy of Medical Education Hospital and Research Institute, Mysuru, IND; 3 Department of Physiology, Farookh Academy of Medical Education Hospital and Research Institute, Mysuru, IND; 4 Department of Biochemistry, Konaseema Institute of Medical Science and Research Foundation, Amalapuram, IND; 5 Central Research Laboratory, Meenakshi Medical College Hospital and Research Institute, Kanchipuram, IND

**Keywords:** diabetes distress, diabetes distress scale-17 (dds-17), emotional burden, rural health centres, type 2 diabetes mellitus

## Abstract

Background: Type 2 diabetes mellitus (T2DM) requires sustained self-management, and diabetes distress is a diabetes-specific psychological burden that can impair coping adherence and outcomes. This study aimed to estimate the prevalence and severity of diabetes distress and evaluate factors associated with distress among adults with T2DM attending rural health centres.

Methods: A community-based cross-sectional study was conducted from March 2025 to December 2025 in rural health centres affiliated with Konaseema Institute of Medical Sciences and Research Foundation, East Godavari District, Andhra Pradesh, India. Adults aged 20-60 years with T2DM were enrolled consecutively (n=390). Diabetes distress was measured using the Diabetes Distress Scale-17 (DDS-17) and classified as no distress (mean item score <2.0), moderate distress (2.0-3.0), and severe distress (>3.0). Prevalence was reported with 95% CIs. Associations with distress categories were tested using chi-squared or Fisher's exact tests. For OR estimation, DDS-17 was dichotomized as any distress (≥2.0) versus no distress (<2.0), and logistic regression was applied.

Results: Among 390 participants, 242 (62.1%) were male patients and 148 (37.9%) were female patients, with a mean age of 40.0 ± 11.6 years. Any diabetes distress was present in 246 participants (63.1%; 95% CI 58.2-67.7), of which moderate distress was seen in 210 (53.8%; 95% CI 48.9-58.7) and severe distress in 36 (9.2%; 95% CI 6.7-12.5). Meanwhile, 144 participants (36.9%; 95% CI 32.3-41.8) reported no distress. Distress categories differed by gender (p<0.00004): among male patients, no distress was reported by 87 (36.0%), moderate distress by 149 (61.6%), and severe distress by six (2.5%); among female patients, no distress was reported by 57 (38.5%), moderate distress by 61 (41.2%), and severe distress by 30 (20.3%). Among distressed participants (n=246), the mean DDS-17 total score was 2.76 ± 0.30. Emotional burden was highest (3.20 ± 0.52), followed by interpersonal distress (2.92 ± 0.21) and regimen-related distress (2.82 ± 0.27), while physician-related distress was lowest (1.41 ± 0.78). On bivariate testing, education, socioeconomic position, marital status, BMI status, and diabetes duration were associated with DDS-17 categories (all p<0.00001). For example, moderate distress was reported in 180 (63.4%) of literate participants (n=284), 100 (55.6%) of middle-class participants (n=180), 150 (48.4%) of married participants (n=310), and 80 (53.3%) of those with 3-6 years of diabetes duration (n=150). It was also found that the combination of metformin and sulfonylurea was associated with higher odds of any distress (OR 2.63; 95% CI 1.48-4.68; p<0.001), and the combination of metformin, sulfonylurea, and statin also showed higher odds (OR 1.93; 95% CI 1.18-3.16; p=0.009).

Conclusions: Diabetes distress was common among rural adults with T2DM, with emotional burden as the dominant domain and severe distress disproportionately concentrated among female patients. Distress severity showed meaningful associations with key sociodemographic and clinical factors, and sulfonylurea-based combination regimens were linked to higher odds of distress. Routine DDS-17 screening with severity-guided and domain-targeted support should be integrated into rural diabetes care, alongside longitudinal studies incorporating glycemic measures, complications, and mental-health covariates to clarify causal pathways.

## Introduction

Type 2 diabetes mellitus (T2DM) is a progressive metabolic disorder that imposes sustained self-management demands and complication risks, creating a clinical setting in which psychological burden can meaningfully influence health behaviors and outcomes [1]. Diabetes distress represents a diabetes-specific emotional state linked to the practical and social pressures of living with diabetes, and it is clinically relevant yet distinct from primary depressive disorders, even though overlap can occur and misclassification may lead to suboptimal care pathways [2]. In South Asia, a recent systematic review and random-effects meta-analysis estimated that diabetes distress affects nearly half of adults with T2DM, with emotional burden emerging as the most frequent domain and substantial between-study variability underscoring the need for locally robust estimates [3]. Evidence from community-based work in India further indicates that clinically significant distress is common and is patterned by sociodemographic and clinical vulnerability, with higher odds reported among female patients, those with lower socioeconomic status, longer diabetes duration, comorbidities, unmet social support needs, and poorer glycemic control, while distressed individuals reported worse perceived health and more unhealthy days [4]. In clinic-based cohorts, clinically significant distress also clusters with treatment complexity and end-organ involvement, aligning with higher hemoglobin A1C (HbA1C), higher BMI, insulin injection burden, nephropathy, and other complications, reinforcing the plausibility that distress and adverse metabolic profiles can reinforce each other in routine care [5]. The present study aimed to estimate the prevalence of diabetes distress and evaluate factors associated with distress among adults with T2DM.

## Materials and methods

A community-based cross-sectional study was conducted between March 2025 and December 2025 among community-residing adults with T2DM who attended rural health centres affiliated with Konaseema Institute of Medical Sciences and Research Foundation, East Godavari District, Andhra Pradesh, India. Eligible participants at the selected centres were enrolled consecutively during the study period. Institutional Ethics Committee approval was obtained (reference no. IEC/PR/2025: 22/04.03.2025).

Participants and eligibility criteria

Adults aged 20-60 years with T2DM were recruited. T2DM was defined according to the American Diabetes Association criteria (ADA) [6] as fasting blood glucose greater than 126 mg/dL, two-hour plasma glucose greater than 200 mg/dL, or HbA1c greater than 6.5%, or by a prior clinician diagnosis with documented ongoing antidiabetic treatment. Exclusion criteria included pregnancy or breastfeeding, concurrent enrolment in another clinical trial, endocrine disorders other than T2DM, malignancy, chronic liver disease, renal failure defined as dialysis or serum creatinine at least or more than 2.0 mg/dL, severe psychiatric illness that could compromise interview reliability, and communication difficulties that could limit valid responses. The sample size was estimated using the single-proportion formula with 95% confidence (Z=1.96), assuming p=0.5 and precision d=0.05, which yielded a required sample of 390 participants.

Data collection and study instruments

Data were gathered using a predesigned semi-structured interview schedule. Diabetes distress was assessed with the 17-item Diabetes Distress Scale (DDS-17) [7], which captures distress experienced during the preceding one month across four domains: emotional burden, regimen-related distress, physician-related distress, and interpersonal distress. Mean item score cutoffs were used to classify distress as no distress (<2.0), moderate distress (2.0 to 3.0), and severe distress (>3.0). Anthropometric and clinical measures, including height, weight, blood pressure, waist circumference, and hip circumference, were recorded using calibrated instruments. When required, a female attendant was present during measurements for female participants.

Statistical analysis

Data were cross-verified, coded, and entered into Microsoft Excel (Microsoft Corp., Redmond, USA) before analysis using IBM SPSS Statistics for Windows version 29.0 (IBM Corp., Armonk, USA). Categorical variables were summarized as n (%) and continuous variables as mean ± SD. DDS-17 was scored as mean item scores and classified as no distress (<2.0), moderate distress (2.0-3.0), and severe distress (>3.0). Prevalence of any distress (≥2.0) and severity levels was reported with 95% CI. Associations between participant factors and DDS-17 categories were tested using Pearson's chi-squared test or Fisher's exact test when expected counts were <5. For odds estimation, DDS-17 was dichotomized (any distress ≥2.0 vs. none <2.0), and univariable binary logistic regression was used to report OR with 95% CI and Wald p-values. Two-tailed p≤0.05 was considered significant.

## Results

A total of 390 adults with T2DM were enrolled. Participant demographics, including sex distribution, mean age, education level, and occupation, are summarized in Table 1.

**Table 1:** Baseline demographic characteristics of participants (n=390)

Characteristic	Category	Total	Male (n=242)	Female (n=148)
Gender, n (%)	Male	242 (62.1)	242 (100.0)	-
	Female	148 (37.9)	-	148 (100.0)
Age (years), mean ± SD	-	40.0 ± 11.6	40.0 ± 11.6	40.0 ± 11.6
Education, n (%)	Literate	284 (72.8)	184 (76.0)	100 (67.6)
	Illiterate	106 (27.2)	58 (24.0)	48 (32.4)
Occupation, n (%)	Self-employed	201 (51.5)	125 (51.7)	76 (51.4)
	Casual labour	105 (26.9)	65 (26.9)	40 (27.0)
	Wage or salaried	64 (16.4)	40 (16.5)	24 (16.2)
	Others	20 (5.1)	12 (5.0)	8 (5.4)

Prevalence and severity of diabetic distress

Among 390 participants, 246 met criteria for diabetic distress on DDS-17, yielding an overall prevalence of 63.1% (95% CI 58.2-67.7). Moderate distress was observed in 53.8% participants (210/390; 95% CI 48.9-58.7) and severe distress in 9.2% participants (36/390; 95% CI 6.7-12.5), while 36.9% had no distress (144/390; 95% CI 32.3-41.8). Among participants with distress, the mean DDS-17 total score was 2.76 ± 0.30 (Table 2).

**Table 2:** Prevalence of diabetic distress by DDS-17 severity DDS-17: Diabetes Distress Scale-17

DDS-17 category	n	%	95% CI
No distress (<2.0)	144	36.9	32.3 to 41.8
Moderate distress (2.0-3.0)	210	53.8	48.9 to 58.7
Severe distress (>3.0)	36	9.2	6.7 to 12.5
Any distress (≥2.0)	246	63.1	58.2 to 67.7

Diabetic distress by gender

DDS-17 distress categories differed by gender (Pearson's chi-squared test, p<0.00004). Among male patients, 36.0% had no distress, 61.6% had moderate distress, and 2.5% had severe distress. Among female patients, 38.5% had no distress, 41.2% had moderate distress, and 20.3% had severe distress (Table 3).

**Table 3:** DDS-17 distress categories by gender The p-value from Pearson's chi-squared test for association between gender and DDS-17 distress categories was <0.00004. DDS-17: Diabetes Distress Scale-17

Gender	No distress (<2.0)	Moderate distress (2.0-3.0)	Severe distress (>3.0)	Total
Male, n (%)	87 (36.0)	149 (61.6)	6 (2.5)	242 (62.1)
Female, n (%)	57 (38.5)	61 (41.2)	30 (20.3)	148 (37.9)

Factors associated with diabetic distress severity

On bivariate analysis, education, socioeconomic position, marital status, BMI status, and duration of diabetes were statistically associated with DDS-17 distress categories. Among literate participants (n=284), moderate distress was observed in 63.4% (n=180) (χ²=92.3032, p<0.00001). Among middle-class participants (n=180), 55.6% (n=100) had moderate distress (χ²=83.6753, p<0.00001). Among married participants (n=310), 48.4% (n=150) had moderate distress (χ²=27.5185, p<0.00001). Among participants with a diabetes duration of 3-6 years (n=150), 80 (53.3%) had moderate distress (χ²=80.3553, p<0.00001). BMI status was also associated with distress categories (χ²=32.5539, p<0.00001) (Table 4).

**Table 4:** Reported bivariate associations with DDS-17 distress categories The p-values were calculated using Pearson's chi-squared test. When any expected cell count was <5, Fisher's exact test was applied. DDS-17: Diabetes Distress Scale-17

Factor (group size)	Key reported cell (moderate distress)	Moderate distress within-group	Test statistic	p-value
Education (literate, n=284)	n=180	63.4%	χ²=92.3032	<0.00001
Socioeconomic position (middle class, n=180)	n=100	55.6%	χ²=83.6753	<0.00001
Marital status (married, n=310)	n=150	48.4%	χ²=27.5185	<0.00001
Duration of diabetes (3-6 years, n=150)	n=80	53.3%	χ²=80.3553	<0.00001
BMI status	Overall association observed; category-wise distribution not reported		χ²=32.5539	<0.00001

DDS-17 domain-wise distress among distressed participants

Among participants with diabetes distress (n=246), emotional burden had the highest mean domain score (3.20 ± 0.52), followed by interpersonal distress (2.92 ± 0.21) and regimen-related distress (2.82 ± 0.27). Physician-related distress had the lowest mean score (1.41 ± 0.78) (Table 5).

**Table 5:** DDS-17 domain scores among distressed participants (n=246) DDS-17: Diabetes Distress Scale-17

DDS-17 domain	Mean ± SD
Emotional burden	3.20 ± 0.52
Regimen-related distress	2.82 ± 0.27
Interpersonal distress	2.92 ± 0.21
Physician-related distress	1.41 ± 0.78

Medication regimens and diabetic distress

Using the dichotomized outcome of any diabetes distress (DDS-17 mean item score ≥2.0) versus no distress (<2.0), univariable logistic regression showed that metformin plus sulfonylurea was associated with higher odds of distress (OR 2.63, 95% CI 1.48-4.68, p<0.001). A combination of metformin, sulfonylurea, and statin was also associated with higher odds of distress (OR 1.93, 95% CI 1.18-3.16, p=0.009) (Table 6).

**Table 6:** Medication regimens associated with any diabetic distress (DDS-17 ≥2.0) Outcome was dichotomized as any diabetes distress (DDS-17 mean item score ≥2.0) versus no distress (<2.0). ORs were calculated from univariable binary logistic regression (Wald test). DDS-17: Diabetes Distress Scale-17

Medication regimen	OR	95% CI	p-value
Metformin + sulfonylurea	2.63	1.48 to 4.68	<0.001
Metformin + sulfonylurea + statin	1.93	1.18 to 3.16	0.009

## Discussion

Key findings of the study

This community-based cross-sectional survey among 390 adults with T2DM attending rural health centres in East Godavari documented a high burden of diabetes distress. Nearly two-thirds of participants met the criteria for any diabetes distress (n=246, 63.1%), followed by moderate distress (n=210, 53.8%), while severe distress affected 9.2% of participants. The gender pattern was clinically important: severe distress clustered strongly among female participants (n=30, 20.3% in female patients vs. n=6, 2.5% in male patients), whereas moderate distress was more frequent in male participants. Domain profiling showed a clear hierarchy of lived burden, with emotional burden scoring highest (mean 3.20), followed by interpersonal and regimen-related distress, while physician-related distress was comparatively low. Distress severity was associated with education, socioeconomic position, marital status, BMI status, and diabetes duration on bivariate testing. Finally, regimen signals emerged: participants on metformin plus sulfonylurea and those on metformin plus sulfonylurea plus statin showed higher odds of any distress, suggesting that treatment complexity and cardiometabolic risk clustering may track with psychological load.

Agreement of findings with existing literature

The observed prevalence falls within the wide South Asian spectrum and supports the view that distress is not a marginal phenomenon in routine diabetes care. A recent South Asian meta-analysis estimated pooled prevalence at 44% with very high heterogeneity and confirmed that emotional burden is typically the dominant domain [3]. The current estimate of 246 (63.1%) is higher than the pooled value, but the direction of domain dominance is concordant, with emotional burden again emerging as the most intense component. This alignment strengthens biological and behavioral plausibility: distress is most intense where the cognitive and emotional labour of self-management is felt most personally, not necessarily where health system contact occurs.

The gender signal also broadly converges with regional evidence that women often experience higher distress intensity. Several clinic and community studies report higher distress among female patients, and particularly stronger emotional burden [4,8,9]. The present data adds nuance: overall, any distress was similar across sexes, but severe distress showed marked female clustering. That pattern matters clinically because severe distress is the category most likely to disrupt adherence, sleep, diet discipline, and follow-up behavior.

Associations with diabetes duration and BMI status are also consistent with the broader literature describing distress as a companion of cumulative disease burden and metabolic strain. Studies in different settings have linked clinically significant distress to poorer glycemic control, higher BMI, and complications, often interpreting distress as both a consequence and amplifier of biomedical risk [5,10]. Even though the present analysis did not incorporate HbA1c, the observed links with BMI and duration point in the same direction: distress tends to cluster where self-management is hardest and where patients sense that the illness is advancing despite effort.

Medication-related findings also align with the psychosocial literature. Distress is associated with treatment modality and perceived regimen load, including medication type and injection burden, and can overlap with concerns about side effects and treatment failure [5,11]. In this study, higher odds of distress among those on metformin plus sulfonylurea (with or without statin) may reflect a pragmatic truth in rural care: intensification often signals rising glycemic difficulty, fear of hypoglycemia, more frequent symptoms, or a sense of being “more ill,” each of which can intensify emotional burden and regimen fatigue.

Disagreement with existing literature and possible explanations

Two areas deserve careful interpretation. First, the overall prevalence of distress in this cohort (63.1%) exceeds several international and some regional estimates, including Vietnam (29.4%) and Romania (25.1% for DDS-17 ≥2), and is also higher than the pooled South Asian estimate [3,12,13]. This is not necessarily a contradiction as much as context. The present cohort was younger (mean age 40 years) than many clinic-based studies, and younger age has been associated with higher distress in some analyses, while older age has sometimes shown lower odds of distress [8,12]. In rural communities, diabetes also collides with wage insecurity, caregiving load, limited transport, and intermittent access to testing supplies. Those stressors are not always captured as “social determinants” in standard models, but they can directly translate into distress scores.

Second, physician-related distress was low in this study, whereas some clinic studies describe more prominent physician or health system-related concerns, including feelings that worries are not taken seriously [14,15]. A plausible explanation is structural rather than interpersonal. Rural primary care interactions can be brief, but they may be culturally familiar and less conflictual, while the heavier strain sits at home: diet negotiation, financial prioritization, and the daily discipline of long-term therapy. This interpretation remains a hypothesis, but it is consistent with the dominance of emotional burden and interpersonal distress in the current domain profile.

Clinical implications

These findings argue for making diabetes distress visible in routine T2DM care rather than treating it as an optional add-on. A simple workflow can be implemented: periodic DDS-17 screening, rapid triage by severity, and domain-targeted counselling. The domain profile suggests where the first gains may lie. Emotional burden was highest, so counselling should focus on coping capacity, fear of complications, perceived failure narratives, and burnout. Interpersonal distress was also high, which supports involving family in education sessions where feasible and addressing social support deficits directly, echoing community evidence linking unmet support needs to distress and poorer perceived health [4,16].

The gender pattern specifically calls for proactive attention to women, not only to address prevalence but also severity. Severe distress in one-fifth of female participants should trigger structured follow-up, screening for depressive symptoms where resources allow, and linkage to counselling or peer support. Importantly, diabetes distress is not synonymous with major depressive disorder, and misclassification can lead to mismatched treatment pathways; practical clinical approaches emphasize distinguishing distress from primary mood disorders while still recognising overlap [2,11]. Medication associations also provide a pragmatic signal: patients on sulfonylurea-based combinations may warrant a brief distress check during visits, especially if hypoglycemia fear, regimen complexity, or perceived escalation is present. Even short, structured conversations can help: clarify goals, normalize difficulty, simplify regimen where safe, and ensure the patient understands the rationale for statin addition as prevention rather than “more disease.”

Strengths and limitations

This study provides a relatively large, community-linked rural estimate using a validated diabetes-specific tool with severity grading and domain-wise profiling. The sample size was adequate for prevalence estimation, and the focus on domain scores adds clinical texture beyond a single pooled prevalence. The analysis also surfaces potentially actionable correlates, including treatment combinations that may mark higher psychosocial load.

Interpretation should remain cautious. The cross-sectional design precludes causal inference and cannot determine whether distress preceded regimen intensification or followed it. Consecutive enrolment from rural health centres affiliated with a single institution may introduce selection bias and limit generalizability to other regions. Several clinically important covariates were not integrated into the association models, particularly HbA1c, documented complications, hypoglycemia history, depression or anxiety symptoms, and structured measures of social support, all of which are repeatedly linked to distress in other settings [5,13,15]. The reported association analysis was primarily bivariate, so residual confounding is likely. Finally, some exposure categories, such as BMI strata distribution within distress levels, were not fully reported, which limits granular interpretation.

## Conclusions

Diabetes distress was highly prevalent among rural adults with T2DM in this cohort, with emotional burden as the dominant domain and a striking concentration of severe distress among women. Distress severity showed significant associations with sociodemographic factors, BMI status, and diabetes duration, while sulfonylurea-based combination regimens were linked to higher odds of distress. Routine distress screening using DDS-17, coupled with severity-guided and domain-targeted support, is clinically justified in rural diabetes care. Future multicentre longitudinal studies that incorporate HbA1c, complications, hypoglycemia history, and mental health measures are needed to clarify causal pathways and identify the most efficient intervention points within resource-constrained settings.
